# Ulcerative dermatitis, tendinitis, tenosynovitis, and desmitis caused by chronic infection with *Serratia odorifera* in a racehorse

**DOI:** 10.1177/10406387261455359

**Published:** 2026-06-21

**Authors:** Carlos O. Schild, Robert Moeller, Paula Yant, Jeff Blea, Javier Asin, Eileen Henderson, Akinyi Nyaoke, Francisco A. Uzal

**Affiliations:** California Animal Health and Food Safety Laboratory System, San Bernardino branches, School of Veterinary Medicine, University of California–Davis, CA, USA; Tulare branches, School of Veterinary Medicine, University of California–Davis, CA, USA; California Animal Health and Food Safety Laboratory System, San Bernardino branches, School of Veterinary Medicine, University of California–Davis, CA, USA; California Horse Racing Board, Arcadia, CA, USA; California Animal Health and Food Safety Laboratory System, San Bernardino branches, School of Veterinary Medicine, University of California–Davis, CA, USA; California Animal Health and Food Safety Laboratory System, San Bernardino branches, School of Veterinary Medicine, University of California–Davis, CA, USA; California Animal Health and Food Safety Laboratory System, San Bernardino branches, School of Veterinary Medicine, University of California–Davis, CA, USA; California Animal Health and Food Safety Laboratory System, San Bernardino branches, School of Veterinary Medicine, University of California–Davis, CA, USA

**Keywords:** desmitis, racehorses, *Serratia odorifera*, tendinitis, tenosynovitis, ulcerative dermatitis

## Abstract

We investigated a case of severe *Serratia odorifera* infection in a racehorse. The horse had developed progressive lameness associated with swelling of the left tarsal region, which progressed to ulcerative dermatitis, tendinitis, tenosynovitis, and desmitis. The plantar side of the superficial digital flexor (SDF) tendon was visible through the skin ulcer, and coalescing nodules were present in the dermis. The horse was euthanized and an autopsy was performed. Grossly, in addition to the changes described above, severe edema of the soft tissues was associated with the ulcer. Microscopically, necrotizing and ulcerative, chronic-active dermatitis with granulation tissue was noted, along with lymphoplasmacytic and suppurative tendinitis (SDF), tenosynovitis (SDF and lateral digital flexor), and desmitis (long plantar ligament and flexor retinaculum [FR]), with collagen degradation and intralesional gram-negative bacteria. *Serratia odorifera* was isolated in pure culture from the ulcerated skin, SDF tendon, FR, and synovial fluid of the tarsus joint. To our knowledge, soft-tissue inflammation caused by *S. odorifera* has not been described in horses previously.

*Serratia odorifera*, *S. marcescens*, and *S. plymuthica* are gram-negative rods in the *Enterobacteriaceae* family.^7^ These *Serratia* spp. are opportunistic agents found in nosocomial infections of immunocompromised human patients.^7,15^ In humans, they have been associated with sepsis (*S. odorifera*, *S. marcescens*),^7,9,15^ endocarditis (*S. odorifera*),^11,14^ liver and lung abscesses (*S. odorifera*, *S. marcescens*, *S. plymuthica*),^8^ infection of the urinary tract (*S. marcescens*),^7^ meningitis, encephalitis (*S. marcescens*),^7^ endophthalmitis (*S. marcescens*),^7^ osteomyelitis (*S. marcescens*, *S. plymuthica*),^3,7^ arthritis (*S. marcescens*),^7^ and skin ulcers (*S. marcescens*).^17^

In domestic animals, only infections with *S. marcescens* have been reported. Affected species include cattle (mastitis, early abortions),^5,6^ horses (septicemia in a foal),^18^ dogs (skin ulceration and abscess),^13^ and cats (endophthalmitis).^10^ A review of PubMed, CAB abstracts, and Google Scholar did not yield any reference to infection by *S. odorifera* in horses. Here, we describe a case of ulcerative dermatitis, tendinitis, tenosynovitis, and desmitis associated with *S. odorifera* infection in a racehorse.

A 5-y-old Thoroughbred racehorse mare with a history of chronic ulcerative dermatitis on the plantar side of the left tarsal and metatarsal region was submitted for autopsy to the California Animal Health Food Safety Laboratory (San Bernardino Branch, San Bernardino, CA, USA). The skin ulcer had grown and failed to heal for ~24 d. The lesion was painful and the mare had hyperesthesia and swelling of the affected area. The horse was treated with enrofloxacin, procaine penicillin G, rifampin, minocycline, flunixin meglumine, hydrocortisone, and phenylbutazone, but given lack of improvement and poor prognosis, she was euthanized.

An autopsy was performed. The left tarsal region was swollen, and a 13.0 × 7.0-cm, deep ulcer was present ~4.0 cm distal to the calcaneal tuber (**Fig. 1A**, **1B**). The ulcer had hemorrhagic edges, and the abundant granulation tissue in the ulcer bed was covered by purulent exudate and occasional crusts. The subcutaneous tissue was firm, yellow, and multinodular. The plantar surface of the superficial digital flexor (**SDF**) tendon was visible through the ulcer, and the epitendon was yellow and partly covered by red nodules and a gelatinous substance (Fig. 1B). The subtendinous calcaneal bursa was replaced by abundant fibrinous exudate, and the long plantar ligament (**LPL**) and the flexor retinaculum (**FR**) were covered by a thick layer of pink, highly vascular soft tissue (**Fig. 1C**).

**Figure 1.**
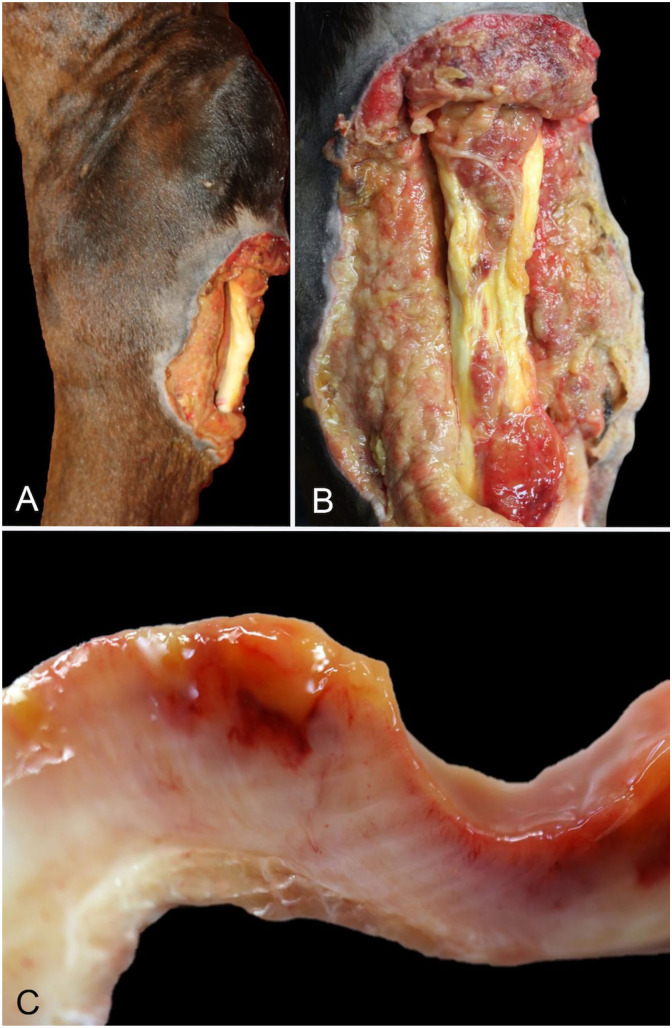
Gross lesions in the left tarsus of a horse with ulcerative dermatitis caused by *Serratia odorifera*. **A.** Lateral view. The superficial digital flexor (SDF) tendon is visible through a skin ulcer. **B.** Plantar view. The palmar side of the SDF tendon is covered by purulent exudate and soft, red tissue. The dermis has multiple nodules. **C**. Transverse section of the flexor retinaculum. The plantar side (top) is expanded diffusely by pink, highly vascular, granulation tissue.

Samples of skin, tendons, ligaments, joint capsule, brain, heart, lungs, liver, kidney, spleen, stomach, and intestines were collected, fixed in 10% neutral-buffered formalin, processed routinely, and 4-µm thick sections were stained with H&E, Gram, and period acid–Schiff/alcian blue (PAS/AB) stains.

Swabs were collected from the skin ulcer, SDF tendon, FR, and synovial fluid of the tarsus joint and inoculated onto trypticase soy agar (TSA) with 5% blood agar, chocolate agar, and MacConkey agar, and incubated aerobically at 37°C for 48 h. Bacterial colonies were identified using MALDI-TOF mass spectrometry (Sirius One; Bruker). *S. odorifera* in pure culture was isolated from the ulcerated skin, SDF tendon, FR, and joint fluid of the tarsus.

Microscopically, the epidermis and dermis of the affected region were ulcerated. The ulcer bed was covered by a layer of viable and degenerate neutrophils, hemorrhage, and bacterial colonies, with an underlying layer of granulation tissue infiltrated by macrophages, lymphocytes, and fewer plasma cells. In the SDF tendon, LPL, and FR, well-demarcated areas of granulation tissue compressed the epi- and endotendon and ligament, and effaced and replaced the fibrils of the collagen bundles (**Fig. 2A**). The granulation tissue was surrounded by a layer of macrophages and neutrophils admixed with immature collagen, fibrin, hemorrhage, karyorrhectic debris, and mixed gram-negative bacteria, which replaced and effaced the lining of the synovial sheath of the SDF tendon (distally) and the subtendinous calcaneal bursa of the SDF tendon (proximally). In less-affected areas, some collagen bundles had lost the normal parallel arrangement of the fibers. Tenocytes and ligamentocytes were absent, accompanied by mild-to-moderate infiltrates of viable and degenerate neutrophils, hemorrhage, and karyorrhectic debris (**Fig. 2B**). The synovial sheath of the SDF and lateral digital flexor tendons had few villi, with the intima thickened by 5–9 layers of synoviocytes admixed with lymphocytes, plasma cells, histocytes, and occasional neutrophils (**Fig. 2C**, **2D**). No significant microscopic lesions were observed in any other tissue.

**Figure 2.**
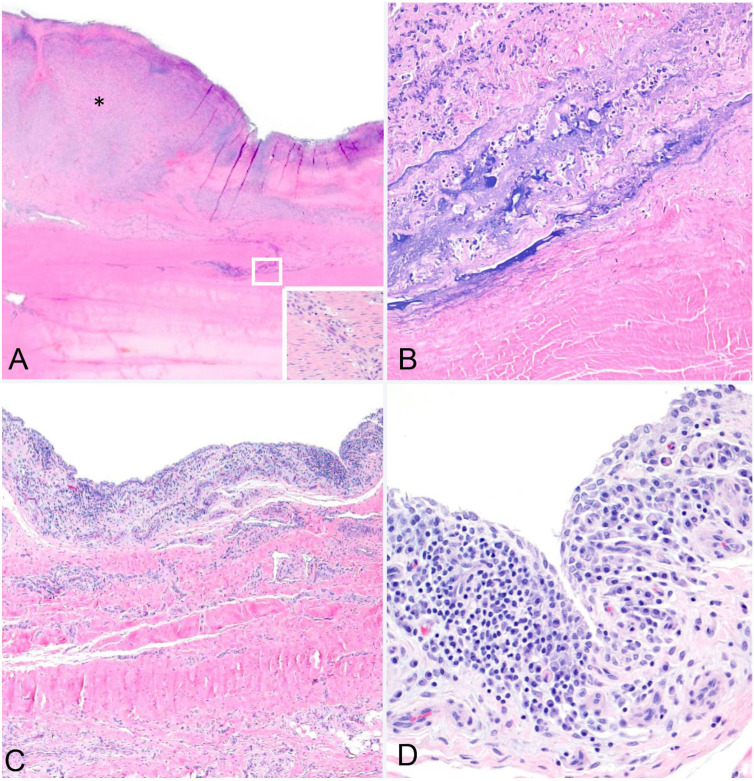
Microscopic lesions in a horse with ulcerative dermatitis, tendinitis, tenosynovitis, and desmitis caused by *Serratia odorifera*. H&E. **A.** Longitudinal section of the superficial digital flexor (SDF) tendon. Focally extensive granulation tissue (asterisk) compresses the epi- and endotendon and replaces the fibers of the collagen bundles. Inset: higher magnification of granulation tissue. **B.** Higher magnification of the rectangle in the SDF tendon in Figure 2A. Note the collagen bundle disarray with loss of normal parallel arrangement of the fibers. Tenocytes are absent, accompanied by moderate numbers of viable and degenerate neutrophils, hemorrhage, and karyorrhectic debris. **C.** Dorsal side of the flexor retinaculum with the synovial sheath of the lateral digital flexor tendon. The synovial intima is expanded by several layers of synoviocytes admixed with lymphocytes and plasma cells that reach the subintima. Collagen bundles are separated by lymphocytes and histiocytes. **D.** Higher magnification of the severe lymphoplasmacytic tenosynovitis in Figure 2C.

The most important findings in our case were ulcerative dermatitis with inflammation of the underlying tendons and other soft tissues, and isolation of *S. odorifera* in pure culture. The lesion in the skin of this horse was similar to those caused by *S. marcescens* in dogs and humans,^13,17^ but to our knowledge, lesions in tendons of horses or other animals associated with *S. odorifera* infections have not been reported previously. Septic tendinitis, tenosynovitis, and/or desmitis associated with *Staphylococcus* spp., *Streptococcus* spp., *Escherichia coli*, and *Enterobacter* spp. have been described in horses, often following a traumatic event and penetrating injury.^2,4,16,20^

*Serratia* spp. are opportunistic bacteria that typically cause infection via the respiratory or urinary tracts. In hospital-acquired systemic infection in humans, the use of contaminated catheters is another important portal of entry.^9,11,14^ Several of these human patients had preexisting conditions or risk factors that contributed to the opportunistic infection of *Serratia* spp., including hematologic disorders, and liver and/or kidney failure.^9,11^ In our case, no evidence of preexisting heart, liver, or kidney failure was evident in the clinical history or during the autopsy, and the portal of entry of *S. odorifera* was not determined. Possible portals of entry include wounds or injection sites. Although this mare had no history of local wounds or injections, we cannot rule out these possibilities.

As in humans, chlorhexidine and benzalkonium-resistant *S. marcescens* can be multidrug resistant and cause opportunistic infections.^1,12^ Virulence factors of *S. marcescens* involved in opportunistic infections include hemolysins, cytotoxic factors such as phospholipase A, and the ability to form biofilms. Antimicrobial resistance (**AMR**) profiles reported in *Serratia* spp. include β-lactam drugs (penicillin, amoxicillin, cephalosporins, carbapenems), aminoglycosides, fluoroquinolone, and trimethoprim–sulfamethoxazole.^7^ In one study of natural antimicrobial susceptibility (**AMS**) of unusual *Serratia* spp. isolates (*S. ficaria*, *S. fonticola*, *S. odorifera*, *S. plymuthica*, *S. rubidaea*), these strains were susceptible to several aminoglycosides, piperacillin, piperacillin-tazobactam, carbapenems, some cephalosporins, fluoroquinolones, and folate pathway inhibitor. The strains were resistant to penicillin G, oxacillin, cefazolin, cefuroxime, macrolides, lincosamides, streptogramins, glycopeptides, fusidic acid, and rifampicin.^19^ In our case, 4 antibiotics were used, including enrofloxacin, procaine penicillin G, rifampin, and minocycline, without significant improvement. In addition to AMR, possible explanations for treatment failure include inappropriate treatment regimen, poor client compliance, repeat contamination, poor perfusion of the infected site, and/or formation of biofilm. Given that this individual horse was received dead for postmortem examination, AMS was not tested, and the strain was no longer available for testing. Should further cases occur, AMS testing should be performed on isolates.

*S. odorifera* should be considered as a potential cause of soft-tissue infections in horses that may be refractory to routine antimicrobial therapy. Culture and AMS testing to direct appropriate therapy may result in a better outcome and would decrease the potential for development of additional AMR in opportunistic bacteria.
